# Severity of hypothyroidism is inversely associated with impaired quality of life in patients referred to an endocrine clinic

**DOI:** 10.1186/s13044-023-00178-0

**Published:** 2023-09-01

**Authors:** Camilla B. Larsen, Kristian Hillert Winther, Per Karkov Cramon, Åse Krogh Rasmussen, Ulla Feldt-Rasmussen, Mogens Groenvold, Jakob Bue Bjorner, Laszlo Hegedüs, Torquil Watt, Steen Joop Bonnema

**Affiliations:** 1 Department of Endocrinology, Odense University Hospital, Kløvervænget 6, DK-5000 Odense C, Denmark; 2 Department of Clinical Research, University of Southern Denmark, Odense, Denmark; 3 Department of Clinical Physiology and Nuclear Medicine, Copenhagen University Hospital Rigshospitalet, Copenhagen, Denmark; 4 Department of Endocrinology and Metabolism, Copenhagen University Hospital Rigshospitalet, Copenhagen, Denmark; 5 Institute of Clinical Medicine, Faculty of Health and Clinical Sciences, Copenhagen University, Copenhagen, Denmark; 6 Department of Palliative Medicine, Bispebjerg Hospital, Copenhagen, Denmark; 7 Department of Public Health, University of Copenhagen, Copenhagen, Denmark; 8 Department of Internal Medicine, Copenhagen University Hospital, Herlev-Gentofte, Denmark

**Keywords:** Hypothyroidism, Autoimmune thyroiditis, Health Related Quality of Life (HRQL), Disease severity, Triiodothyronine (T3), Levothyroxine (LT4) substitution

## Abstract

**Purpose:**

We investigated the association between health-related quality of life (HRQL) and the severity of hypothyroidism at diagnosis in patients referred to a secondary hospital clinic.

**Methods:**

Sixty-seven adult patients referred from primary care were enrolled. All patients had newly diagnosed hypothyroidism due to autoimmune thyroiditis and were treated with levothyroxine (LT4). The dose was adjusted according to thyroid function tests aiming at a normal plasma thyrotropin. Patients were stratified according to the severity of hypothyroidism in two different ways: the conventional approach (subclinical or overt hypothyroidism) and a novel approach according to the change (decrease or increase) in plasma level of free triiodothyronine index (FT3I) following LT4 treatment. The ThyPRO-39 questionnaire was used for measurement of HRQL at referral to the Endocrine Outpatient Clinic (higher score corresponds to worse HRQL).

**Results:**

Free thyroxine index (FT4I) at diagnosis correlated positively with the scores on the Hypothyroid Symptoms and Tiredness scales (*p* = 0.018 for both). In accordance, patients with subclinical hypothyroidism (*n* = 36) scored higher on Hypothyroid Symptoms (*p* = 0.029) than patients with overt hypothyroidism (*n* = 31). The difference in HRQL was more pronounced if patients were stratified according to the dynamics in FT3I following LT4 treatment. Thus, patients who showed a decrease in FT3I following treatment (*n* = 24) scored significantly worse for Anxiety (*p* = 0.032) and Emotional Susceptibility (*p* = 0.035) than patients with an increase in FT3I (*n* = 43).

**Conclusion:**

Patients referred to an endocrine clinic with mild hypothyroidism had an impaired HRQL, compared to patients with more severe hypothyroidism. The most likely explanation of this finding is a lower threshold for seeking medical consultation and secondary care referral if HRQL is deteriorated. The dynamics in plasma FT3I following treatment may be more sensitive for such a discrimination in HRQL than a stratification according to the thyroid function tests at diagnosis.

**Supplementary Information:**

The online version contains supplementary material available at 10.1186/s13044-023-00178-0.

## Introduction

The standard treatment of hypothyroidism caused by autoimmune thyroiditis (AIT) is life-long levothyroxine (LT4) substitution [1]. However, current treatment regimens may be inadequate, as a substantial fraction of patients have persistent symptoms and a reduced health-related quality of life (HRQL) [2–7]. Whether a correlation exists between HRQL and the thyroid hormone plasma levels within the normal range is controversial, and non-hormonal factors are also involved [8–12].

There are numerous reports of HRQL in patients with AIT and hypothyroidism [2, 7, 9, 13, 14], but only few studies have investigated the relationship between HRQL and the severity of the disease at diagnosis. In a previous study, we found no significant difference in HRQL between patients with subclinical and overt hypothyroidism, either at baseline or during LT4 treatment [2]. While it is well established that LT4 substitution has a profound effect on health and well-being in patients with severe hypothyroidism, the effect of therapy in more subtle thyroid dysfunction is a source of debate [7]. Thus, in a randomized trial, older individuals with subclinical hypothyroidism showed no benefit in HRQL from LT4 treatment, compared with placebo [15].

When euthyroid, defined as normalized plasma thyrotropin (TSH), many patients on LT4 substitution show relatively low levels of plasma triiodothyronine (T3) [16–18]. Gullo et al. reported that approximately 15% of patients on LT4 treatment had a plasma T3 below the normal reference range [19]. Usually, the severity of hypothyroidism is rated according to the plasma levels of TSH or thyroxine (T4) at diagnosis. However, a stratification of severity of hypothyroidism according to the dynamic in plasma T3 after initiation of LT4 substitution might theoretically represent a complementary approach [20]. With progressive hypothyroidism, the conversion of T3 is upregulated due to increased deiodinase 2 (DIO2) activity [21]. Eventually, plasma T3 declines as the thyroid failure progresses, despite increased DIO2 activity. A reversal of these pathways follows when LT4 therapy is initiated, often leading to a decrease in plasma T3 among patients diagnosed with mild hypothyroidism [16–18, 22]. In patients with more severe hypothyroidism, plasma T3 increases by LT4 treatment, although levels within the reference range are not always attained [19–21]. Based on such an approach, not used in any previous study, patients with hypothyroidism may be stratified into mild and moderate-to-severe thyroid failure, respectively, according to the plasma T3 response following LT4 treatment.

In a prospective study of patients referred to an endocrine clinic, we investigated the association between HRQL and the severity of hypothyroidism defined in two different ways, i.e. the conventional approach according to the plasma levels of TSH and T4 at diagnosis (subclinical or overt hypothyroidism, respectively) and a complementary—and novel—approach according to the dynamic in plasma T3 after initiation of LT4 (decrease or increase, respectively).

## Methods

### Study design and participants

Patients ≥ 18 years with a diagnosis of hypothyroidism due to AIT and initiation of LT4 substitution within the last three months (newly diagnosed), were recruited at the Endocrine Outpatient Clinic, Odense University Hospital, Denmark, in the period May 2014 through January 2020. Patients were referred by their general practitioner or another hospital unit. The diagnosis of AIT was based on an anti-thyroid peroxidase (TPO) antibody level > 100 IU/mL at diagnosis. The main exclusion criteria were: a previous diagnosis of toxic nodular goiter or Graves’ disease, postpartum thyroiditis, previous radioiodine therapy or thyroid surgery, pregnancy, severe comorbidity, immunomodulatory medication, or other medication known to affect thyroid function. All patients gave written informed consent. At diagnosis (before HRQL assessment), and according to standard clinical guidelines, LT4 treatment was initiated, with subsequent dose adjustment at intervals of 5–6 weeks until plasma TSH was within the reference range.

The study was embedded in the multicenter CATALYST trial [23]. This randomized placebo-controlled multicenter trial examines the effect of selenium supplementation in AIT. The study protocol, published previously [23], was approved by the Regional Scientific Ethical Committees for Southern Denmark (project ID: S-20130123) and registered at ClinicalTrials.gov (ID: NCT02013479). Inclusion of more than 400 patients in the CATALYST trial, 117 of whom had newly diagnosed AIT (diagnosis of AIT within the last three months), was completed by January 2020. For the purpose of the present study, we restricted the study population to patients enrolled at Odense University Hospital (*n* = 67) as access to the electronic medical record was required to retrieve biochemical data.

### Biochemical analyses

From the electronic medical files biochemical values at diagnosis and at euthyroidism were retrieved. All samples were analyzed in the same laboratory by the same methods throughout the study period. Plasma TSH (reference level: 0.3–4.0 mIU/L), total T3 (1.3–2.2 nmol/L), total T4 (60–130 nmol/L) and T4 uptake test (0.64–1.22) were measured by a two-site chemiluminescent immunometric assay using Cobas® 8000 (Roche Diagnostics, U.S). Free T3 index (FT3I) and free T4 index FT4I were calculated based on the T4 uptake test. Anti-TPO antibodies were determined using AutoDelfia (Perkin Elmer, Waltham, MA). The coefficient of variation (CV) was 8.3% and 4.2% at TSH levels of 0.084 and 11.3 mIU/L, respectively; CVs were 3.3% and 2.9% at total T3 levels of 1.9 and 4.2 nmol/L, respectively; CVs were 4.6% and 4.5% at total T4 levels of 78 and 168 nmol/L, respectively and CV for T4 uptake was 3.5% at T4 uptake level of 0.976.

### Stratification according to severity of hypothyroidism

Patients were stratified into two groups according to the severity of hypothyroidism. This was done in two different ways. In one approach, the traditional, patients were grouped into overt hypothyroidism (plasma TSH above the reference range and free T4 index (FT4I) below the reference range) or subclinical hypothyroidism (plasma TSH above and FT4I within the reference ranges) at diagnosis.

In another approach, the patients were stratified into group A and group B according to the change in FT3I plasma level following LT4 treatment. The FT3I at diagnosis was compared with the FT3I level when a normal plasma TSH was achieved, typically within 2–4 months. Thus, group A comprised patients showing a decrease in plasma FT3I, while group B included those with an unaltered or increased plasma FT3I following treatment with LT4.

### Health related quality of life

HRQL was measured at the Endocrine Outpatient Clinic, Odense University Hospital, in relation to inclusion in the CATALYST trial, and before initiation of the CATALYST trial medication. LT4 treatment was initiated prior to the HRQL assessment, most often by the general practitioner when the diagnosis of hypothyroidism was established. We used the validated short-form of the disease-specific thyroid-related Patient-Reported Outcome questionnaire (ThyPRO-39) [24–27]. This questionnaire consists of 39 items on physical, mental, and social domains of functioning and wellbeing in hypothyroidism, hyperthyroidism, non-toxic goiter, and thyroid eye disease. The items employ a recall period of four weeks and are summarized in 10 multi-item scales as well as a single item scale measuring overall impact of thyroid disease on quality of life. The items are scored from 0 to 4, following a Likert scale (where “0” is equivalent to “nothing at all” and “4” to “very much”). The average score of items in each scale is divided by four and multiplied by 100 to yield scores from 0 to 100, with higher scores indicating poorer health status. A ThyPRO composite score, based on 21 items from the tiredness, cognition, anxiety, depressivity, emotional susceptibility, impaired social life, and impaired daily life scales, plus the overall quality of life item, was also computed. For specific items, please see the entire ThyPRO-39 questionnaire (Supplementary ThyPRO questionnaire).

### Statistical analysis

Due to deviation from the normal distribution, values of TSH and anti-TPO were log-transformed before calculation. Groups were compared using chi-squared test or Student’s *t-*test for parametric data, and Mann–Whitney U test for nonparametric data. Data are presented as frequencies or means ± standard deviation (SD). For evaluation of the ThyPRO questionnaire Student’s *t-*test was used as well as the “minimal important change” (MIC) according to Nordqvist et al. [28]. MIC is defined as the smallest change that patients perceive as important. MIC differs between the ThyPRO scales, being in the range 6.3–14.3 for groups and 8.0–21.1 for individuals [28].

A backward stepwise regression and simple correlations were used for testing associations between variables. The analyses were performed using SPSS Statistics, version 25 (IBM, Armonk, NY). Statistical significance was defined as *p* < 0.05. All statistical tests were two-sided.

## Results

### Patient characteristics

Sixty-seven patients were included. Characteristics of the patients, when stratified into subclinical (*n* = 36) and overt hypothyroidism (*n* = 31), are shown in Table 1. Patients with subclinical hypothyroidism had a higher body weight than those with overt hypothyroidism (82.1 ± 13.6 vs. 72.2 ± 12.8 kg; *p* = 0.003).

**Table 1:** Patient characteristics at diagnosis and at euthyroidism when stratifying into subclinical and overt hypothyroidism

	Subclinical *n* = *36*	Overt *n* = *31*	*p*-value
Age at diagnosis (years)	46.6 ± 14.5	52.8 ± 14.4	0.086
Weight at HQRL assessment (kg)	82.1 ± 13.6	72.2 ± 12.8	0.003*
Gender (n)			
Male	6	7	
Female	30	24	0.542
Intake of LT4 (n)			
Morning	4	7	
Bedtime	32	24	0.206
Brand of LT4 (n)			
Eltroxin®	31	25	
Euthyrox®	5	6	0.547
Dose of LT4 at euthyroidism	99.2 ± 35.7	103.0 ± 38.7	0.674
Smoker at diagnosis (n)			
Never	12	10	
Smoker	6	3	
Ex-smoker	18	18	0.846
Time to euthyroidism (days)	114 ± 85	116 ± 67	0.885
LogTSH at diagnosis^a^	0.91 ± 0.25	1.18 ± 0.39	0.002*
Anti-log TSH, mU/L	8.1	15.1	
Total T3 at diagnosis, nmol/L	1.70 ± 0.31	1.48 ± 0.29	0.004*
Total T4 at diagnosis, nmol/L	78.67 ± 15.3	54.9 ± 10.9	< 0.001*
T4 uptake at diagnosis	1.08 ± 0.16	1.09 ± 0.13	0.811
FT4I at diagnosis, nmol/L^a^	72.7 ± 8.5	50.8 ± 9.5	0.002*
FT3I at diagnosis nmol/L	1.59 ± 0.22	1.38 ± 0.28	< 0.001*
FT4I/logTSH at diagnosis	85.2 ± 23.7	49.4 ± 20.6	< 0.001*
FT4I/FT3I ratio at diagnosis	46.5 ± 6.3	37.6 ± 8.2	< 0.001*
LogTSH at euthyroidism^a^	0.28 ± 0.24	0.33 ± 0.27	0.52
Anti-log TSH, mU/L	1.9	2.1	
Total T3 at euthyroidism, nmol/L	1.61 ± 0.26	1.49 ± 0.24	0.073
Total T4 at euthyroidism, nmol/L	101.9 ± 18.2	89.6 ± 15.2	0.004*
T4 uptake at euthyroidism	1.06 ± 0.15	1.00 ± 0.07	0.060
FT4I at euthyroidism, nmol/L^a^	96.7 ± 13.9	89.5 ± 15.5	0.49
FT3I at euthyroidism nmol/L	1.53 ± 0.21	1.50 ± 0.22	0.546
FT4I/FT3I ratio at euthyroidism	63.9 ± 9.65	61.1 ± 12.8	0.310
LogAnti-TPO at diagnosis	2.80 ± 0.53	2.84 ± 0.53	0.768
Anti-log anti-TPO, kIU/L	630	691	
Time from diagnosis to HRQL assessment (days)	72 ± 53	70 ± 49	0.891

*Abbreviations: HRQL* health-related quality of life, *LT4* levothyroxine, *TSH* thyrotropin, *T3* triiodothyronine, *T4* thyroxine, *FT3I* Free T3 index, *FT4I* Free T4 index, *Anti-TPO* thyroid peroxidase antibodies

^*^P-value < 0.05. Statistically significant difference is highlighted in bold

^a^In agreement with the stratification (subclinical/overt hypothyroidism)

Characteristics of the patients stratified according to the change in FT3I plasma level following treatment are shown in Table 2. Patients in group A (*n* = 24) were younger than in group B (*n* = 43); 44.6 ± 15.1 vs. 52.2 ± 13.8 years (*p* = 0.047). As expected, patients in group B were biochemically more hypothyroid at diagnosis than those in group A, reflected by differences in the thyroid function tests (Table 2). No other significant differences were found at diagnosis between group A and group B. At euthyroidism following treatment, a significant difference between the two groups was found in the FT4I/FT3I ratio; 66.5 ± 11.9 in group A vs. 60.4 ± 10.3 in group B (*p* = 0.043). The LT4 dose at time of euthyroidism was insignificantly lower in group A than in group B (Table 2).

**Table 2:** Patient characteristics at diagnosis and at euthyroidism for group A (decrease in plasma FT3I after initiation of levothyroxine) and group B (increase or no change in plasma FT3I after initiation of levothyroxine)

	Group A *n* = *24*	Group B *n* = *43*	*p*-value
Age at diagnosis (years)	44.6 ± 15.1	52.2 ± 13.8	0.047*
Weight at HQRL assessment (kg)	78.9 ± 13.7	76.8 ± 14.3	0.570
Gender (n)			
Male	7	6	
Female	17	37	0.131
Intake of LT4 (n)			
Morning	3	8	
Bedtime	21	35	0.734
Brand of LT4 (n)			
Eltroxin®	23	32	
Euthyrox®	2	10	0.186
Dose of LT4 at euthyroidism	96.5 ± 30.7	103.4 ± 30.1	0.44
Smoker at diagnosis (n)			
Never	9	13	
Smoker	4	5	
Ex-smoker	11	25	0.753
Severity of hypothyroidism at diagnosis (n)			
Subclinical	18	18	
Overt	6	25	0.011*
Time to euthyroidism (days)	125 ± 102	110 ± 58	0.492
LogTSH at diagnosis	0.94 ± 0.26	1.09 ± 0.39	0.071
Anti-log TSH, mU/L	8.7	12.3	
Total T3 at diagnosis, nmol/L	1.69 ± 0.33	1.55 ± 0.31	0.091
Total T4 at diagnosis, nmol/L	70.25 ± 18.1	66.22 ± 19.3	0.38
T4 uptake at diagnosis	1.03 ± 0.15	1.12 ± 0.13	0.017*
FT4I at diagnosis, nmol/L	68.2 ± 12.2	59.5 ± 14.4	0.011*
FT3I at diagnosis nmol/L	1.66 ± 0.22	1.40 ± 0.25	< 0.001*
FT4I/ logTSH at diagnosis	78.7 ± 27.7	63.01 ± 27.7	0.031*
FT4I/FT3i ratio at diagnosis	41.8 ± 8.6	42.7 ± 8.6	0.693
LogTSH at euthyroidism	0.27 ± 0.30	0.33 ± 0.23	0.47
Anti-log TSH, mU/L	1.9	2.1	
Total T3 at euthyroidism, nmol/L	1.51 ± 0.29	1.58 ± 0.23	0.323
Total T4 at euthyroidism, nmol/L	98.9 ± 19.0	94.72 ± 17.2	0.371
T4 uptake at euthyroidism	1.05 ± 0.16	1.02 ± 0.09	0.496
FT4I at euthyroidism, nmol/L	95.2 ± 16.9	92.4 ± 14.0	0.51
FT3I at euthyroidism nmol/L	1.55 ± 0.21	1.45 ± 0.24	0.098
FT4I/FT3i ratio at euthyroidism	66.5 ± 11.9	60.4 ± 10.3	0.043*
LogAnti-TPO at diagnosis	2.79 ± 0.57	2.84 ± 0.51	0.927
Anti-log anti-TPO at diagnosis, kIU/L	616	692	
Time from diagnosis to HRQL assessment (days)	66 ± 48	74 ± 52	0.499

^*^P-value < 0.05. Statistically significant difference is highlighted in bold. For abbreviations, see Table 1

### Health related quality of life

**Fig. 1:**
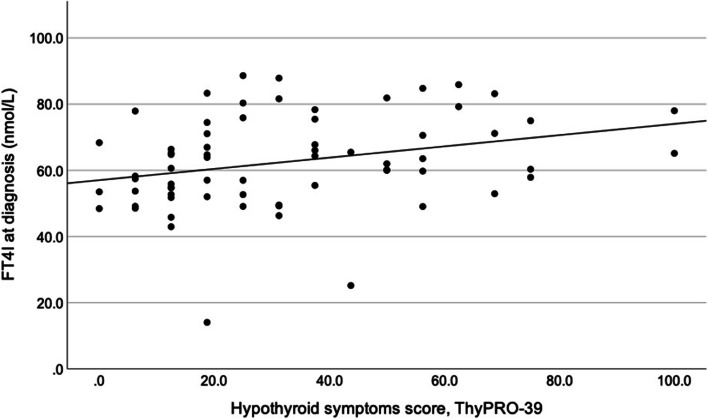
Correlation between Free T4 index (FT4I) at diagnosis and the ThyPRO-39 Hypothyroid Symptoms score in (*r* = 0.289; *p* = 0.018). Higher score indicates more symptoms

**Fig. 2:**
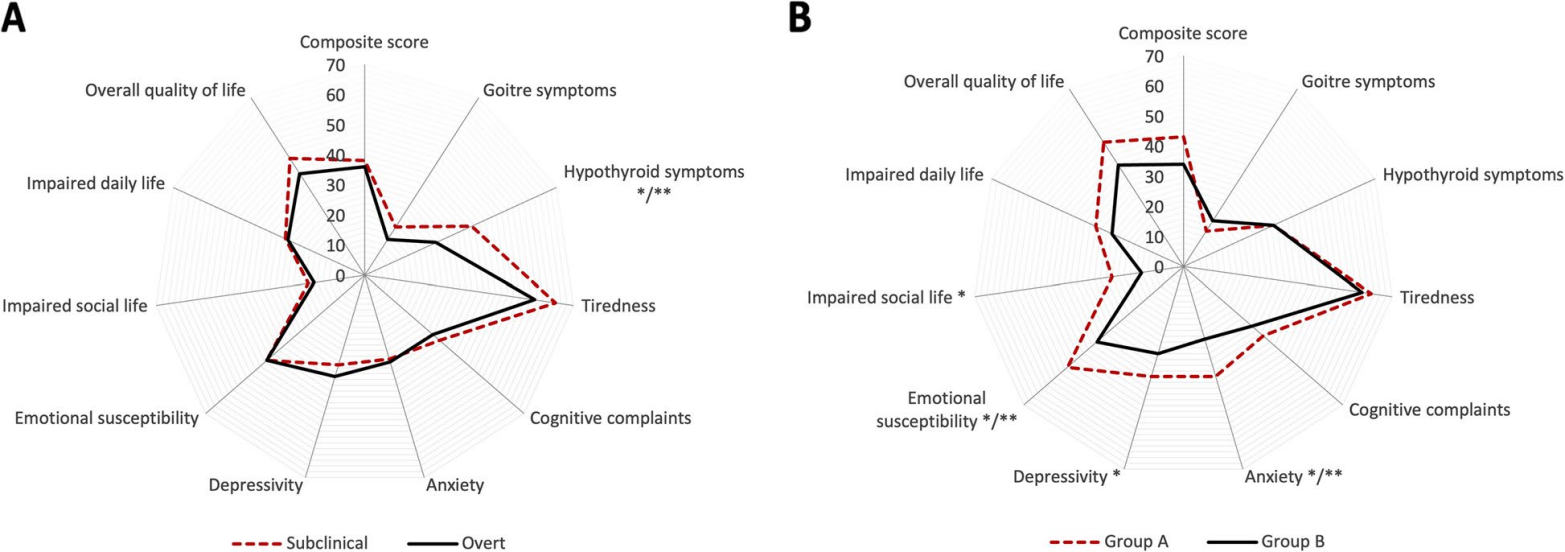
**A** Radar plot showing ThyPRO-39 scale scores for subclinical (*n* = 36) and overt hypothyroidism (*n* = 31) at diagnosis. **B** Radar plot showing ThyPRO-39 scale scores for group A (decrease in plasma FT3I after initiation of levothyroxine, *n* = 24)) and group B (increase or no change in plasma FT3I after initiation of levothyroxine, *n* = 43) Each scale ranges 0–100, with higher scores indicating poorer quality of life. Difference exceeding minimal important change (MIC) are marked with *. Difference with *p*-value below 0.05 are marked with **

FT4I at diagnosis correlated positively with the scores on the Hypothyroid symptoms scale (Fig. 1) and the Tiredness scale (*r* = 0.289 and *r* = 0.243, respectively, *p* = 0.018 for both). Accordingly, patients with subclinical hypothyroidism scored significantly higher on the Hypothyroid Symptoms scale than patients with overt hypothyroidism (*p* = 0.029, Table 3 and Fig. 2).

**Table 3:** Comparison of ThyPRO scale scores between subclinical and overt hypothyroidism and comparison of ThyPRO scale scores between group A (decrease in plasma FT3I after initiation of levothyroxine) and group B (increase or no change in plasma FT3I after initiation of levothyroxine)

ThyPro scale scores	**Subclinical vs overt hypothyroidism**				**Dynamics in FT3I following treatment**			
	*Subclinical* *n* = *36*	*Overt* *n* = *31*	Difference (Sub—overt) [95% CI]	*p*-value	*Group A* *n* = *24*	*Group B* *n* = *43*	Difference A vs B [95% CI]	*p*-value
Composite score	37.8 ± 19.7	36.3 ± 18.3	1.5 [-7.8 to 10.9]	0.741	42.7 ± 19.9	33.9 ± 17.8	8.7 [-1.2 to 18.5]	0.084
Goitre symptoms	18.6 ± ± 18.5	13.7 ± 10.8	4.9 [-2.4 to 12.2]	0.186	13.9 ± 16.6	17.6 ± 14.9	-3.7 [-11.9 to 4.5]	0.155
Hypothyroid symptoms	38.7 ± 24.8	26.0 ± 21.8	**12.7**^**a**^ [**1.3 to 24.2**]	**.0290**	32.6 ± 22.4	32.9 ± 25.3	-0.4 [-12.5 to 11.6]	0.818
Tiredness	64.1 ± 23.6	56.8 ± 23.1	7.4 [-4.1 to 18.7]	0.202	62.5 ± 24.8	59.7 ± 22.9	2.7 [-9.6 to 15.1]	0.519
Cognitive complaints	32.9 ± 24.3	29.9 ± 24.2	3.0 [-8.9 to 14.8]	0.615	34.5 ± 21.9	29.9 ± 25.3	4.5 [-7.4 to 16.3]	0.343
Anxiety	28.9 ± 22.9	30.0 ± 19.9	-1.1 [-11.5 to 9.4]	0.841	38.1 ± 24.8	24.5 ± 17.9	**13.5**^**a**^ [**3.1 to 24.0**]	**0.032**
Depressivity	31.2 ± 19.2	34.9 ± 17.5	-3.8 [-12.7 to 5.2]	0.404	38.3 ± 21.4	29.9 ± 16.0	8.3^a^ [0.8 to 17.5]	0.110
Emotional susceptibility	42.6 ± 24.2	43.1 ± 20.0	-0.5 [-11.3 to 10.3]	0.925	50.7 ± 22.3	38.4 ± 21.1	**12.3**^**a**^ [**1.4 to 23.5**]	**0.035**
Impaired social life	18.9 ± 22.1	16.7 ± 20.2	2.3 [-8.0 to 12.5]	0.662	24.3 ± 24.9	14.3 ± 17.9	9.9^a^ (0.6 to 20.5]	0.126
Impaired daily life	28.5 ± 24.9	28.2 ± 24.9	0.3 [-11.9 to 12.5]	0.957	32.0 ± 24.6	26.4 ± 24.8	5.6 [-6.9 to 18.3]	0.379
Overall quality of life	45.8 ± 32.5	40.3 ± 30.1	5.5 [-9.7 to 20.7]	0.473	48.9 ± 32.5	40.1 ± 30.4	8.8 [-7.5 to 25.1]	0.297

Mean ± SD ThyPRO scale scores (0–100, higher scores indicating worse HRQL)

Statistically significant difference is highlighted in bold

^a^Difference exceeding MIC (minimal important change) according to Nordqvist et al. [28]

As compared with the former stratification (subclinical and overt hypothyroidism), the differences in HRQL were more pronounced if patients were stratified according to the FT3I dynamics, as shown in Table 3, and illustrated in Fig. 2. For the majority of items, patients with a decline in FT3I (group A) following treatment had higher ThyPRO scores (i.e. worse HRQL), compared to those showing an increase in FT3I (group B). Differences exceeding MIC (minimal important change) [28], were found for Anxiety, Depressivity, Emotional Susceptibility, and Impaired Social Life. Significant differences between group A and group B were found for Anxiety (*p* = 0.032) and Emotional Susceptibility (*p* = 0.035).

In a regression analysis, Anxiety and Emotional Susceptibility correlated negatively with age (β-coeff. = -0.275; *p* = 0.024, and β-coeff. = -0.304; *p* = 0.012, respectively; Table 4). The decrease in plasma FT3I correlated positively with plasma FT3I at diagnosis and Anxiety (β-coeff. = 0.446; *p* < 0.001, and β-coeff. = 0.264; *p* = 0.022, respectively; Table 5).

**Table 4:** Key variables included in the regression analysis for prediction of anxiety and emotional susceptibility

**All variables included in the equation**	**Beta Coeff** (Anxiety)	***P***	**Beta Coeff** (Emotional susceptibility)	***P***
Gender (male/female)	0.133	0.372	0.321	0.026
Age at diagnosis (years)	-0.274	0.048	-0.313	0.018
Weight at HQRL assessment (kg)	0.083	0.563	0.157	0.248
LT4 intake (morning/at bedtime)	-0.316	0.029	-0.200	0.139
Smoking	0.124	0.358	0.227	0.079
Time to euthyroidism (days)	0.156	0.259	0.260	0.049
LogAntiTPO	0.065	0.626	-0.077	0.538
FT4I at diagnosis	-0.487	0.560	-0.780	0.324
FT3I at diagnosis	0.480	0.386	0.554	0.290
FT4I/logTSH at diagnosis	0.122	0.780	0.714	0.087
FT4I/FT3i ratio at diagnosis	0.592	0.343	0.443	0.452
LogTSH at diagnosis	0.376	0.264	0.729	0.024
Brand of LT4 (Eltroxin® or Euthyrox®)	-0.301	0.031	-0.217	0.096
Time from diagnosis to HRQL assessment (days)	-0.035	0.798	-0.085	0.504
**By backward stepwise regression**				
Age at diagnosis (years)	-0.275	0.024	-0.304	0.012

*Abbreviations: HRQL* health-related quality of life, *LT4* levothyroxine, *Anti-TPO* thyroid peroxidase antibodies, *TSH* thyrotropin, *T3* triiodothyronine, *T4* thyroxine, *FT3I* Free T3 index, *FT4I* Free T4 index

**Table 5:** Key variables included in the regression analysis for prediction of a decrease in plasma FT3I after initiation of levothyroxine therapy

All variables included in the equation	Beta-Coefficient	*P*
Gender (male/female)	-0.033	0.850
Age at diagnosis (years)	-0.215	0.179
Weight at HQRL assessment (kg)	-0.033	0.850
LT4 intake (morning/at bedtime)	0.010	0.952
Smoking	-0.021	0.883
Time to euthyroidism (days)	0.114	0.456
LogAntiTPO at diagnosis	0.025	0.861
FT4I at diagnosis	-0.196	0.819
FT3I at diagnosis	0.430	0.441
FT4i/logTSH at diagnosis	0.249	0.590
FT4I/FT3I ratio at diagnosis	0.281	0.658
LogTSH at diagnosis	0.036	0.921
Brand of LT4 (Eltroxin® or Euthyrox®)	-0.087	0.566
Time from diagnosis to HRQL assessment (days)	-0.027	0.841
Composite score	0.851	0.542
Goitre score	-0.148	0.377
Hypo score	-0.095	0.604
Tiredness score	-0.478	0.168
Cognition score	-0.256	0.488
Anxiety score	0.105	0.773
Depression score	0.044	0.870
Emotional susceptibility score	-0.192	0.547
Social score	-0.177	0.667
Daylife score	0.161	0.521
Overall Quality of Life score	-0.056	0.708
**By backward stepwise regression**		
FT3I at diagnosis	0.446	< 0.001
Anxiety	0.264	0.015

Dependent variable is FT3I decrease

*Abbreviations: HRQL* health-related quality of life, *LT4* levothyroxine, *Anti-TPO* thyroid peroxidase antibodies, *TSH* thyrotropin, *T3* triiodothyronine, *T4* thyroxine, *FT3I* Free T3 index, *FT4I* Free T4 index

According to the exclusion criteria, patients with major comorbidities and those receiving drugs with impact on the thyroid were excluded from the study. Six patients received estrogen containing drugs; excluding these individuals did not change our results (data not shown).

## Discussion

In this study, we investigated the association between the severity of hypothyroidism and HRQL in newly diagnosed patients. A few previous studies found that ThyPRO scores correlate with the thyroid hormone levels, even within the normal range [9, 10], and with the level of thyroid autoantibodies [11]. Usually, graduation of the thyroid failure, for example into overt and subclinical hypothyroidism, is based on plasma levels of TSH and T4. To our knowledge, our study is the first to include the dynamics in plasma FT3I following LT4 treatment as a marker of perceived disease severity. In line with clinical observations, the dynamics in plasma FT3I reflected the extent of the thyroid failure. Thus, patients showing a decrease in FT3I following LT4 treatment (group A) had significantly higher FT4I at diagnosis compared with those showing an increase in plasma FT3I (group B). The difference between the two groups in the T3 dynamics is most likely due to adaptive differences in the DIO2 activity, as described above.

The main finding of our study is that patients with less pronounced hypothyroidism had significantly higher ThyPRO scores (i.e. worse HRQL) than those with biochemically more severe disease. Thus, FT4I at diagnosis correlated positively with ThyPRO scores in two scales. Depending on how patients were stratified according to disease severity, different items were affected, such as Hypothyroid Symptoms, Anxiety, Emotional Susceptibility, and Tiredness. Patients with subclinical hypothyroidism had a higher body weight than those with overt hypothyroidism, which potentially could have affected HRQL. However, this was not the case when patients where stratified based on the FT3I dynamics. Further, body weight was adjusted for in the regression analysis.

It is a paradox should patients with mild hypothyroidism have a worse HRQL than those with more severe disease. Therefore, we speculate that our finding may be due to selection bias, meaning that patients with a lower threshold for symptoms are being referred to a secondary center at an early stage of the disease. Most probably, such patients also have a gap in HRQL compared to the background population. A stratification of disease severity based on the FT3I dynamics showed more pronounced differences in HRQL than a conventional stratification into subclinical and overt hypothyroidism. This indicates that a stratification of hypothyroid patients by the former method may offer a more physiological approach, at least in the context of HRQL among newly diagnosed patients referred to an endocrine clinic. Potentially, the two ways of stratification may be complementary, as they seem to discriminate various items of ThyPRO-39 differently (Fig. 2).

Up to 80% of intracerebral T3 is derived from local conversion from T4 catalyzed by DIO2 [16, 29, 30], and it has been speculated that genetic variants of this enzyme are involved in the mental and cognitive symptoms characterizing some patients with AIT despite normal plasma TSH on LT4 substitution [16, 29, 30]. However, the majority of randomized placebo-controlled clinical trials comparing LT4 with LT4 + liothyronine (LT3) combination therapy failed to demonstrate any significant difference in HRQL between the two treatment regimens [7, 8, 29, 31]. Nevertheless, many patients treated for hypothyroidism have focus on T3 plasma levels, supported by ongoing media debates. Some patients may even have noticed that their plasma T3 level has declined after starting LT4 substitution therapy. In case of persistent fatigue or cognitive dysfunction, without any improvement by LT4 substitution, such patients may conclude that their symptoms are caused by the drop in plasma T3, thus requesting another treatment, e.g. LT4 + LT3 combination therapy. An important observation in our study—and of clinical relevance—is that patients with a decline in plasma T3 after LT4 treatment suffered from impaired HRQL. The difference in the dynamic in plasma T3 between group A and group B was most probably due to differences in the severity of hypothyroidism at diagnosis. If the two groups had been equally hypothyroid, the differences in HRQL and the plasma T3 dynamics might be more directly and causally related.

In contrast to most previous studies of patients with hypothyroidism, we used the extensively validated and disease specific ThyPRO-39 for measuring patient-reported outcomes [24]. In addition, the study was performed in a controlled clinical setting, including both men and women across a wide age range, and no other thyroid diseases than autoimmune thyroiditis were included. Limitations also exist. First, the study was not primarily designed to perform cross-sectional investigations of HRQL in relation to thyroid function tests. Thus, a sample size calculation was not performed specifically for the present study. However, we find that the risk of the study being statistically underpowered is low, as the patient subgroups differed significantly in several of the specified outcomes. Second, the patients had initiated LT4 treatment (within the last three months) before they filled-in the ThyPRO questionnaire. Eleven out of 67 patients had obtained biochemical euthyroidism when HRQL was assessed; however, excluding these individuals from the analysis did not significantly change the results (data not shown). Had the HRQL been assessed exactly at the time of diagnosis in all patients, the HRQL might have been more affected, potentially resulting in an even stronger correlation between HRQL and disease severity. Third, being enrolled in the CATALYST trial, half of the patients received 200 ug selenium per day, or placebo, respectively, in addition to the LT4 substitution. It can be argued that selenium could have influenced the T4 to T3 conversion and thus the plasma T3 level. However, we previously showed, in a large, randomized placebo-controlled trial, that selenium supplementation had no effect on either plasma FT3 level or the plasma FT3/FT4 ratio [32]. Fourth, although the coefficient of variation of measurement of T3 was at the same level as that of T4, T3 is more prone to fluctuations than T4 due to the influence of non-thyroidal illness [33, 34]. However, we consider these factors to be of minor relevance as individuals with major comorbidities and those receiving drugs with impact on the thyroid were excluded from the study. Further, excluding the six patients on estrogen containing drugs did not change our results. Finally, HRQL was assessed only initially and not after stable euthyroidism was achieved. Thus, it is unknown whether the two groups of patients, independent of the method used for stratification, achieved a similar HRQL after being euthyroid for a longer period.

In conclusion, in this study population, patients with more severe hypothyroidism at diagnosis had better HRQL compared to patients with milder hypothyroidism. The difference in HRQL was more pronounced if patients were stratified based on the FT3 dynamics following LT4 therapy as compared to the conventional approach, i.e. subclinical or overt hypothyroidism based on thyroid function tests at diagnosis. Our findings may reflect selection bias with an early referral of vulnerable patients to a secondary care center, where an initial decrease in plasma T3 is a common biochemical response to LT4 treatment.

### Supplementary Information

**Additional file 1.**

## Data Availability

The datasets generated during and/or analyzed during the current study are available from the corresponding author on reasonable request.

## Abbreviations

AIT: Autoimmune thyroiditis
Anti-TPO: Thyroid peroxidase antibodies
FT4I: Free thyroxine index
FT3I: Free triiodothyronine index
HRQL: Health-related quality of life
LT4: Levothyroxine
MIC: Minimal important change
TSH: Thyrotropin
T4: Thyroxine
T3: Triiodothyronine
ThyPRO-39: Thyroid-related Patient-Reported Outcome questionnaire
